# Unpredictable singleton distractors in visual search can be subject to second-order suppression

**DOI:** 10.3758/s13414-025-03028-3

**Published:** 2025-02-26

**Authors:** Brandi Lee Drisdelle, Alon Zivony, Martin Eimer

**Affiliations:** 1 School of Psychological Science, University of Bristol, Beacon House, Queens Road, Bristol, BS8 1QU UK; 2 School of Psychology, University of Sheffield, Sheffield, UK; 3 School of Psychological Sciences, Birkbeck, University of London, London, UK

**Keywords:** Visual search, Visual attention, Attentional suppression, Letter-probe paradigm, Attentional capture, Second-order suppression

## Abstract

Recent evidence suggests that attentional capture by salient-but-irrelevant distractions can be avoided via suppression, thereby improving performance in visual search. Initial evidence suggested it is only possible to suppress salient distractors with constant and predictable features (first-order suppression). We show that previous failures to find evidence for second-order suppression of unpredictable feature singletons may have been due to low feature variability: If it is probable that the salient distractor colour is the target colour on another trial, suppressing this item might hinder performance. We first validated a new multiframe letter-probe paradigm, where observers counted the search displays with a target shape and always reported as many letter probes as possible from the final display. When target and singleton colours were constant (Experiment 1), a singleton suppression effect was observed, with probe letters at the singleton distractor location reported less frequently than those at non-singleton distractor locations. When two randomly swapped target/singleton colours were employed (Experiment 2), no suppression effect was observed, replicating previous findings. Critically, when target-colour items and the singleton could have one of eight different random colours (Experiment 3), a robust suppression effect reappeared. These observations demonstrate that first-order suppression is not universal, and that second-order suppression can be applied to singleton distractors under some circumstances. Suppression effects were observed for displays with and without targets, suggesting that they are not a product of direct target-singleton competition.

**Supplementary Information:**

The online version contains supplementary material available at 10.3758/s13414-025-03028-3.

## Introduction

In everyday life, we often find ourselves distracted by salient events in our environment. For example, when driving, a flashing advertisement or shop sign can capture our gaze. It therefore seems intuitive to assume that salient information will always automatically attract our attention, even when such information is irrelevant or even detrimental to our current goals. However, the question about whether and when we can and cannot resist attentional capture by salient distractors continues to remain a matter of vigorous debate (Luck et al., 2021). According to stimulus-driven accounts of attention, salient events automatically attract attention regardless of their task relevance (Jonides & Yantis, 1988; Theeuwes, 1991, 1992; Wang & Theeuwes, 2020). Conversely, according to goal-dependent accounts, our visual system can prioritise goal-related visual information (Folk & Remington, 1998; Folk et al., 1992) and can therefore at least sometimes avoid distraction by irrelevant events. A core assumption of goal-dependent models is that capture only occurs if the salient objects possess at least one feature relevant to our current task. Thus, we might pay attention to a flashy advertisement if one of its features (e.g., its colour) is related to our goals, but are able to avoid it otherwise. Despite decades of research, evidence supporting both accounts continues to emerge (i.e., stimulus-driven: Wang & Theeuwes, 2018, 2020; goal-dependent: Folk & Remington, 2015; Turatto et al., 2018). Current research has therefore started to assess conditions that determine whether we can or cannot resist attentional capture by salient but irrelevant signals.

A recently proposed hybrid model, the *signal suppression hypothesis*, includes elements of both the stimulus-driven and goal-dependent accounts (Gaspelin & Luck, 2018a; Gaspelin et al., 2015; Sawaki & Luck, 2010, 2011). According to this model, bottom-up signals that can trigger capture are automatically generated by salient stimuli regardless of their relevance, in line with stimulus-driven accounts. However, in line with goal-dependent accounts, these signals can be suppressed if they do not match an active task set, resulting in attentional capture being prevented. Evidence supporting this model comes from studies of ocular movements (Adams et al., 2023; Blakley et al., 2022; Gaspelin et al., 2017, 2019), psychophysics (Chang & Egeth, 2019; Gaspelin et al., 2015), event-related potentials (Drisdelle & Eimer, 2021, 2023; Gaspar et al., 2016; Gaspelin & Luck, 2018b; Sawaki & Luck, 2010; Stilwell et al., 2022; van Moorselaar et al., 2021), and single-unit recordings in monkeys (Cosman et al., 2018). Perhaps the most compelling behavioural evidence comes from the *letter-probe paradigm*, developed by Gaspelin and colleagues (Gaspelin et al., 2015). In this task, *search trials* are interleaved with infrequent *probe trials*. On search trials, participants look for a shape-defined target stimulus. Distractor stimuli are either all non-singletons (distractors that are the same colour as the target), or, on a subset of trials, also include a singleton object (a salient different-coloured distractor). On probe trials, letters are briefly superimposed on all search items and participants must report as many letters as possible. These probe letter reports provide information about how attention is allocated to each location in the search display. The key finding, the *suppression effect*, is that letters were less likely to be reported when they appear at the location of the singleton distractor than when they are presented at the location of one of the other non-singleton distractors. This effect was interpreted as direct evidence for the active suppression of distractor singleton locations, which is triggered to prevent attentional capture. More recently, this letter-probe paradigm has been modified to investigate distractor suppression with larger set sizes (e.g., Stilwell & Gaspelin, 2021), and to evaluate effects of intertrial priming (Wirth et al., 2023).

### Mechanisms of suppression

Although there is mounting evidence that information provided by statistical regularities and the spatial distribution of singleton distractors can minimize their interference during visual search (Ferrante et al., 2018; Huang et al., 2022; Leber et al., 2016; Liesefeld & Müller, 2021; Theeuwes et al., 2022; van Moorselaar et al., 2021, 2023; Wang & Theeuwes, 2018), the exact mechanisms that result in distractor suppression are still a matter of debate (Gaspelin & Luck, 2018b; Liesefeld et al., 2021, 2022; Ma & Abrams, 2023a, b; Sauter et al., 2018; Won & Geng, 2018). One critical question is whether suppression is always linked to particular features (e.g., a specific colour; *first-order suppression*; Gaspelin & Luck, 2018b; Vatterott et al., 2018), or whether it can also be based more generally on feature discontinuities, regardless of their specific value (i.e., any colour discontinuity; *second-order suppression*; Won et al., 2019*)*.

Using the letter-probe paradigm, Gaspelin and Luck (2018b) observed a suppression effect (i.e., reduced probe report accuracy at the location of the singleton relative to non-singleton distractor locations) only when the colours of the singleton distractor and search items (target and non-singleton distractors) remained constant throughout the experiment. In contrast, when the singleton and search item colours were changed unpredictably between trials (preventing suppression based on a specific colour), no evidence of probe suppression emerged (see also Graves & Egeth, 2015; Vatterott & Vecera, 2012). They concluded that attentional suppression of a singleton depends on foreknowledge of its feature values – such as a specific colour – and cannot be applied to a dimension or a feature-blind representation – such as all uniquely coloured distractors (regardless of its specific colour). In other words, observers can apply first-order but not second-order suppression.

However, some recent observations appear inconsistent with this conclusion. Ma and Abrams (2023a) examined probe suppression in a task where participants no longer reported a unique target shape, but instead whether squares or circles were more prevalent in a search display (e.g., for displays that contained three squares, two circles, and one hexagon, squares would be the correct answer). In some displays, the hexagon was a colour singleton, and the colours of the singleton and search items were swapped unpredictably across trials. Critically, Ma and Abrams (2023a) did observe a suppression effect (i.e., fewer reports of probe letters superimposed on hexagons when these items were colour singletons than when they were not), even when only two colours were used, so that either colour were equally likely to be relevant or irrelevant on any given trial. They argued that the absence of second-order suppression observed by Gaspelin and Luck (2018b) was a result of the fact that participants had to find a specific shape-defined target. This may have encouraged search for unique features, which might in turn have prevented the suppression of colour singleton distractors.

However, an alternative explanation for the results reported by Ma and Abrams (2023a) is that their modified paradigm could have motivated observers to attend to all search display items in the task-relevant colour (including the hexagon on singleton-absent trials) to determine the most frequent shape. This would increase the probability of reporting probes the location of the nominally task-irrelevant hexagon, and thus invalidate the difference between reports of probes at non-singleton versus singleton distractor locations – the critical comparison for assessing suppression effects.

Another line of evidence for second-order suppression comes from studies which instead promote singleton capture (i.e., the additional-singleton paradigm; Theeuwes, 1991, 1992), where participants search for a singleton-shape target among homogenous shapes that are the same colour as the target. The classic observation is that the presence of a singleton-colour distractor increases reaction times (RTs), indicating that this item captured attention. Using such a paradigm, Won et al. (2019) varied the frequency of singleton presence (20% vs. 80% of trials) and singleton colour, which was either fixed or changed randomly among four possible colours. They showed that increasing the frequency of the singleton decreased the likelihood of capture (i.e., it reduced the distractor interference on RTs), which could suggest that observers were able to avoid frequent singletons at least to some extent. Importantly, this effect was present even when singleton colour was variable, indicating that this suppression could be applied when the colour of the singleton was not predictable (see also Vatterott et al., 2018). One possibility is that in the case of Gaspelin and Luck (2018b), the small number of possible colours (two), and the fact that they employed fixed target/distractor pairings when the number of possible colours was increased to four, might have prevented suppression due to the high probability of any colour being the target. This might lead to all colours being treated as potential targets, eliminating second-order suppression as an efficient search strategy. Thus, second-order suppression may still be available when the number of task-irrelevant singleton colours is further increased. Alternatively, it is also possible that because the colour of the target remained constant in Won et al. (2019), participants could use a colour-specific target template, which may have been employed to counteract attentional capture by singleton distractors specifically when these singletons were frequent.

To conclude, it is unclear which aspect of Gaspelin and Luck’s (2018b) paradigm prohibited suppression of singleton distractors when their colour was unpredictable. Was it the need to search for unique and specific feature-defined targets, the small number of colours, the fact that target and distractor colours swapped across trials, or some combination of factors? Since the letter-probe paradigm has become highly popular for studying suppression, answering this question is crucial for future research on the topic, and the goal of the current study was to provide such an answer.

**Fig. 1:**
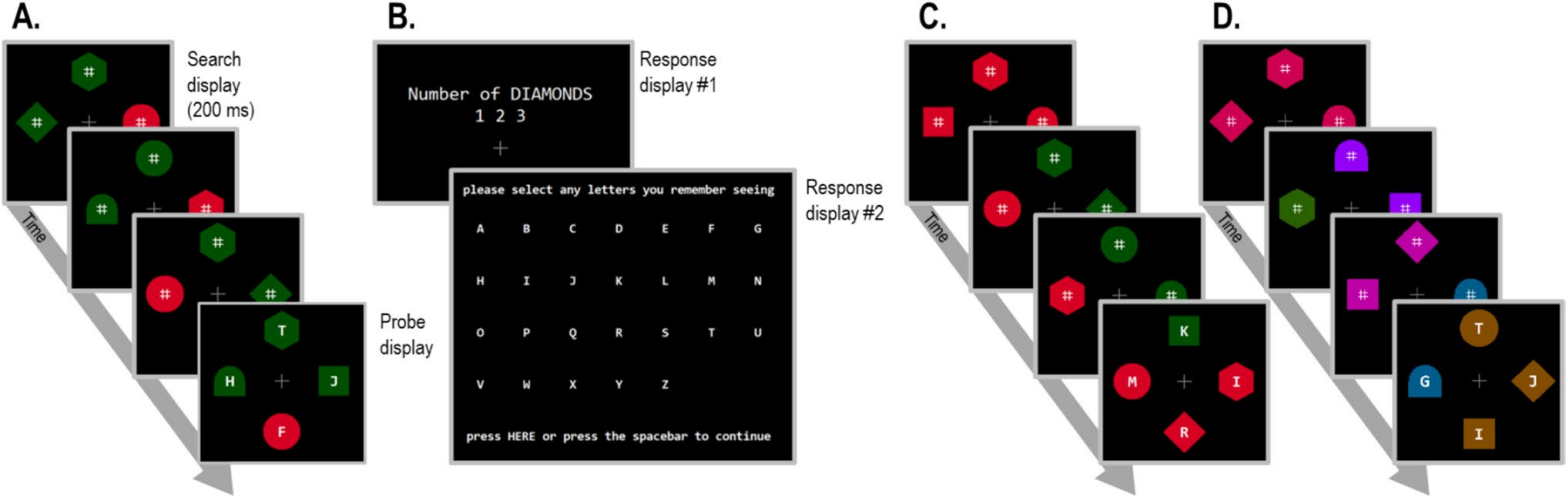
Illustration of experimental procedure in Experiment 1 (Fig. 1A), response prompts for all experiments (Fig. 1B),Experiment 2 (Fig. 1C),and Experiment 3 (Fig. 1D). Each display was presented for 200 ms separated by a 500-ms blank display with fixation. The target in all the examples portrayed here is a diamond. The colour of the target and singleton distractor was constant in Experiment 1 (Fig. 1A), randomly switched between two colours in Experiment 2 (Fig. 1C), and randomly chosen from eight possible colours in Experiment 3 (Fig. 1D). Probe displays were preceded by two to four search displays and were followed by two response displays (Fig. 1B). The primary *counting task* response prompt was displayed first, and the secondary *probe task* response prompt was displayed second

We employed a version of the letter-probe paradigm where successful search requires the identification of a unique shape-defined target (unlike Ma & Abrams, 2023a) that was modified to emphasize the importance of distractor suppression by presenting several search displays including irrelevant colour singletons in rapid succession (*multiframe letter-probe paradigm*). On each trial, a sequence of three to five four-item search displays was presented (including both target-present and target-absent displays). At the end of each sequence, participants reported the number of shape-defined target items. In the final display, probe letters were superimposed on each shape, and participants also reported all letters that they were able to recall (see Fig. 1). In each display, a singleton distractor was present. Experiment 1 was designed to validate this new letter-probe paradigm by replicating the original suppression effect observed by Gaspelin et al. (2015), under conditions where singleton distractor colours remain constant. Experiment 2 and Experiment 3 then employed the same paradigm, but now with variable and hence unpredictable singleton distractor colours.

In Experiment 2, two colours were swapped between search items (target and non-singleton distractors) and the singleton distractor, as in Gaspelin et al. (2018b) and Ma and Abrams (2023a). In Experiment 3, eight equiluminant colours were used, and the target/non-singleton distractor and singleton distractor colours were determined at random on each trial. If second-order distractor suppression is generally unavailable, a suppression effect should only be observed in Experiment 1 but not in Experiments 2 and 3. The same pattern of results should be found if first-order suppression is the only option when searching for a unique target feature, as suggested by Ma and Abrams (2023a). If second-order suppression was available even when target-defining features are unique but could not be applied in Gaspelin and Luck (2018b) because there were only two colours or colour pairs that swapped between target and singleton distractor items, a suppression effect should emerge in Experiment 3 but not in Experiment 2. Finally, the fact that the final display on each trial could either include a target item or not made it possible to investigate whether any distractor suppression effects were affected by the presence of a target in the same display.

## Experiment 1

Experiment 1 was designed to validate a new version of the letter-probe paradigm, the *multiframe letter-probe paradigm*. Each trial consisted of a sequence of three to five four-item displays, where colours of search items and the singleton distractor were held constant, followed by two response prompts. On the final display, a probe letter was presented on each shape. Participants reported (A) the number of displays with a pre-defined target shape and (B) the number of letters they recall from the final display. Our goal was to replicate the suppression effect, where probe accuracy at the singleton distractor location is lower than probe accuracy at the non-singleton distractor locations. This new design has several advantages over the original letter-probe paradigm.

First, the multiple frame procedure allows us to present a probe display on each trial while still ensuring that participants are searching for the target, as observers must attend to every display to count the number of target-present displays. Importantly, they cannot predict when the final probe display will occur because the total number of displays varies randomly across trials (three to five). This paradigm has the potential to increase the number of probe trials, improving the signal-to-noise ratio and thus the robustness of the data.^1^ Second, to further increase the data points available to compute probe suppression effects, we presented a task-irrelevant singleton distractor on every display. While it has been argued that distractor-present and distractor-absent trials should be presented with equal probability for the singleton to be considered distracting (Wöstmann et al., 2022), distractor suppression effects have been observed when distractors are more probable (e.g., Ma & Abrams, 2023a, b). Finally, this new paradigm also included probe displays without a target in the display, allowing us to observe whether suppression effects were modulated by the presence versus absence of direct competition between the target and the singleton distractor. Because prior studies measuring probe suppression effects typically only included target-present trials, this question has not yet been tested empirically. In all three experiments reported here, a target was present or absent in 50% of probe displays.

### Methods

The procedure for this experiment and subsequent experiments was approved by the departmental ethics committee at Birkbeck, University of London.

#### Sample size selection

Sample size for Experiment 1 and subsequent Experiments were based on Gaspelin et al. (2015), where the critical *t*-test of Experiment 2 showed that probe letters at the singleton distractor location were reported less frequently than those at the non-singleton distractor locations (*d*_*z*_ = 0.95). To replicate this suppression effect with a power of 0.95 and an alpha of 0.05, a sample of 17 participants would be required (Faul et al., 2007). The required sample size was doubled, and 34 participants were in the final sample of all experiments. This decision was made for two reasons. First, we wanted sufficient power to measure any effects of target presence (an additional factor) and any interactions. Also, previous research has shown mixed results regarding whether the suppression effect exists in experimental designs with unpredictable colours, meaning that these effects might be weaker than when the colour is held constant.

#### Participants

Thirty-six (36) participants completed Experiment 1. Two participants were removed due to having near chance accuracy on the main task, leaving 34 participants in the final sample (age: *M* = 26.2 years; *SD* = 10.4 years; 24 women and 10 men). All participants kept for final analysis had a mean accuracy within 2.5 standard deviations from the overall mean on the target counting task (*M* = 75.5%, *SD* = 15.4%).

#### Stimuli and procedure

The task was conducted online and distributed using E-prime Go 1.0, the online platform for E-prime 3.0 software. Participants were asked to complete the task in a quiet area with curtains closed, away from bright light, and to put their mobile devices on silent mode to avoid any distractions during the experiment. We also requested that participants position themselves at an arm’s length distance from the monitor. Once the programme was initiated, participants answered demographic questions (age and gender) and reported their monitor size (in inches) if it was known. This question could also be skipped, and participants were instead asked to fit a credit card (ID-1-sized card as defined by ISO/IEC 7810) to a rectangle on the screen which would determine monitor size. The size of the rectangle could be adjusted (using keys 4 and 6 to decrease and increase the size, respectively) until it was the same size as the card. Participants were then presented with the consent form, where they gave consent by pressing the Y key or aborted the experiment using the Esc key.

As their main task, three to five search displays were presented, and the objective was to report the number of displays with a predefined target shape (*counting task*). A second task was also completed: On the final search display, letters were briefly presented at the centre of each of the shapes, and participants reported the letters they remembered (*probe task*). The response display for the counting task appeared after the trial sequence and was followed by the response display for the probe task. Combining these tasks ensured that participants were performing visual search (reflected by accuracy in the counting task) and assessed the processing of each location in the probe display (probe task).

Each trial began with a fixation cross presented for 900 ms. Search displays were each presented for 200 ms, with an inter-trial interval of 500 ms. Each display contained four shapes, one above fixation, one below fixation, and two lateral to fixation (one to the left and one on the right; see Fig. 1). All shapes in displays before the probe display had hash (#) symbols presented at the centre to ensure that the letters in the final display were not recognized due to low-level discrepancies (i.e., the inclusion of a character on top of the four shapes). The letters in the probe displays were presented on each of the four shape centres for the first 150 ms (the shapes were presented for 200 ms) and followed by a 500-ms blank display before the counting task response prompt appeared. Participants first reported the number of displays that contained a predefined target item (one to three displays out of three to five displays) using a keyboard. The number of targets and displays within each trial were determined randomly. Once a response was registered, the 26 letters of the alphabet were displayed on the screen, and participants reported how many letters they remembered seeing on the final search display using either a keyboard or a mouse. When the mouse was used, participants selected letters by pressing on an area within an invisible 0.8° × 1.0° rectangle around it. Once pressed, a (0.8° radius) circle appeared around the selected character to provide participants with visual feedback that their response was registered. Similarly, when the keyboard was used, a circle was traced around the letter corresponding to the key press to indicate that it had been selected.^2^ Participants could report up to four (the total number of shapes) letters. When participants were satisfied with the letters selected (and this was below the maximum number of letters), they pressed the spacebar, and the next trial began automatically after 800 ms. If four letters were reported, the trial continued automatically. Figure 1 illustrates the time course of a single trial.

Half of the trials had a target present on the final probe display (and the other half did not). The number of displays, the location of the search objects (target when present, singleton and non-singletons), and the shapes of the distractors themselves (non-singleton and singleton) were determined randomly. Items were presented on a black background, with a grey fixation cross at the centre of the screen. The centre of each shape was at approximately 2° from fixation.^3^ The target could be a diamond (1.6° × 1.6°) or a circle (0.7° in radius; counterbalanced between-subjects). The other distractors (singleton and non-singleton) items in the display could be a square (1.13° × 1.13°), a gate stimulus (1.2° × 1.2°), a hexagon (1.5° × 1.5°), or the other target shape that did not serve as the target for that subject (i.e., if the target was a diamond, then the circle would be a distractor, and vice versa). Every item in the display was a unique shape. There were two possible colour schemes (counterbalanced between subjects): red (RGB: 215, 0, 35) search items (target and non-singleton distractors) with a green (RGB: 0, 79, 0) singleton distractor, or green search items with a red singleton distractor. All different types of search displays were presented in random order, with the constraint that half of the probe displays had a target present.

There were 100 experimental trials, separated into five blocks of 20 trials each. The experiment began with three separate practice blocks. In the first two practice blocks, both tasks (first the counting task and then the probe task) were practiced separately. Participants completed a minimum of two trials per task but were given the option to perform additional trials. Trial-by-trial feedback was given for the counting task during the first practice block. A third practice block of 20 trials where participants performed both tasks together was then completed. Participants could repeat this final practice block as well. Block-by-block feedback on the average accuracy in the counting task was provided for the third practice block and all experimental blocks.

#### Analysis

For the counting task, overall accuracy was generally high (*M* = 0.75, *SD* = 0.15, ranging from 0.43 to 0.96), meaning that participants were attending to the shapes in the display and identifying the target correctly above chance level (0.33). The majority of incorrect responses were trials where participants miscounted the number of targets by one (e.g., they reported seeing one target when there were in fact two). Trials where participants miscounted by two targets (i.e., if there were three targets but participants reported one target, or vice versa) were removed from the final analysis (1.8% of all trials). Whereas a correct response indicates that participants likely identified all targets, an incorrect response does not indicate a failure to discriminate target presence and absence in all displays (i.e., participants could have identified one or two targets in a series of displays). Assuming independence of response choice across displays, the approximate accuracy of a participant for a trial with three displays is proportional to the third root of overall accuracy (assuming the probability of cancelling errors is low). Thus, an overall trial accuracy rate of 0.43 (the lowest accuracy) would still correspond to 0.75 accuracy rate for individual displays, and this corresponding rate would be higher for trials with more displays. For this reason, we retained trials where participants miscounted the number of targets by one for the final analysis.

For the probe task, we calculated the proportion of correctly identified letters (probe accuracy) on the target (when present), the non-singleton distractors, and the singleton distractor separately. These three separate averages allowed us to directly assess if attention was allocated to each type of object differently. For the non-singleton distractors, this was calculated by dividing the sum of the correctly identified non-singleton shape probes by the number of actual non-singleton shape probes (two in target-present probe displays and three in target-absent probe displays).

Inferential statistical analyses were conducted on the probe task data. To compare probe type means, analyses of variance (ANOVAs) were conducted and followed up with *t*-tests when appropriate. If the sphericity assumption was violated, *p* values were adjusted using Greenhouse–Geisser corrections, with the original uncorrected degrees of freedom reported.

Some tests reported in this experiment and the following experiments include the interpretation of null results. Therefore, statistical tests with theoretically important non-significant results were supplemented with a corresponding calculation of a Bayes factor in favour of the null hypothesis (*BF*_*01*_). All tests were conducted using the anovaBF and lmBF functions from the BayesFactor package in R (Morey & Rouder, 2023). As recommended by van Doorn et al. (2023), we used the “maximal” model (i.e., the model with both participant intercepts and effect slopes as random effects) to evaluate our effects, although all the results were comparable when only participant intercepts were included as random factors. Bayes factors associated with a two-way interaction were calculated by dividing two Bayes factors: (i) the Bayes factor associated with the main effect for both factors and the interaction term, and (ii) the Bayes factor associated with the model that includes only the two main effects. Since we had no a priori expectations regarding these effects, we used the default medium prior (*r* = 0.50), yet in all experiments we obtained similar results with wider priors (*r* = 0.707 or *r* = 1.0). We consider a *BF*_*01*_ to provide evidence for the null hypothesis if it is larger than 3 (i.e., *BF*_10_ < 0.33). Such a finding indicates that, given the data, we should update our belief (relative to our prior belief) in favour of the null hypothesis by a factor of 3.

### Results

Participants reported an average of 2.15 letters per probe frame, and 1.64 (76%) of these letters were present in the probe array. Participants reported a roughly equal number of correct letters in target-present and target-absent probe displays (present: 1.62 letters, absent: 1.66 letters, *t*(33) = 1.19, *p* = 0.24, *d*_*z*_ = 0.20, *BF*_*01*_ = 8.55).

**Fig. 2:**
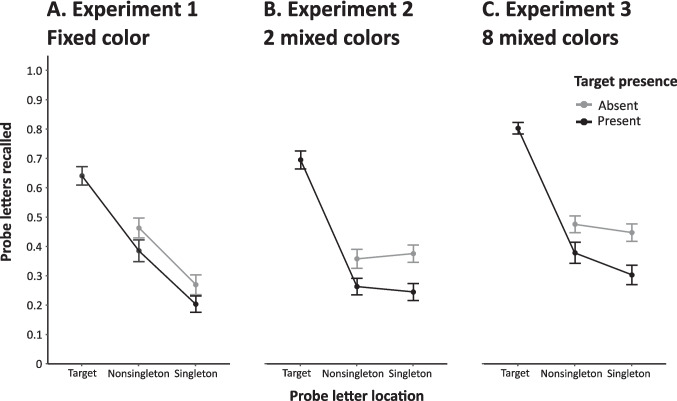
Proportion of probe reports by location in Experiments 1–3. Proportion of correctly reported probes as a function of whether the probes appeared on the target location, the non-singleton distractor locations, or the singleton distractor location. Results are presented separately for probe displays with and without a target. In Experiment 1 (A) and Experiment 3 (C), letters presented at the singleton distractor location were reported the least frequently and letters presented at the target location the most frequently. In Experiment 2 (B), there was no difference in the reporting frequency of letters presented in the non-singleton distractor and singleton distractor locations. Error bars represent the standard error of the mean for each condition

As shown in Fig. 2A, when a target was in the display, probe letters at the target location were more frequently reported (*M* = 0.64, *SE* = 0.03) than those at either the singleton (*M* = 0.20, *SE* = 0.03) or non-singleton (*M* = 0.39, *SE* = 0.04) distractor locations. A one-way ANOVA on target-present trials comparing the proportion of accurately reported letters at all three locations (target, singleton distractor, non-singleton distractors) confirmed that there was a significant difference between conditions, *F*(2, 66) = 56.30, *p* < 0.001, ${\eta }_{p}^{2}$ = 0.63. Follow-up *t*-tests (two-tailed; corrected for multiple comparisons) showed that the letter presented at the target location was reported more frequently than letters at distractor locations (non-singleton: *t*(33) = 6.77, *p* < 0.001, *d*_*z*_ = 1.16; singleton: *t*(33) = 9.4, *p* < 0.001, *d*_*z*_ = 1.61). This result demonstrates that the probe task is a sensitive measure of attention allocation to objects (see also Gaspelin et al., 2015). Critically, letters presented in the non-singleton distractor locations were more frequently reported than those presented at the singleton distractor location, *t*(33) = 4.6, *p* < 0.001, *d*_*z*_ = 0.79. We therefore replicated the suppression effect observed by Gaspelin et al. (2015).

We also examined whether suppression of the singleton distractor depended on the target’s presence. Figure 2A suggests that the likelihood of reporting the letter in the singleton distractor location was reduced compared with the non-singleton location for both target-present and target-absent trials. We conducted a repeated-measures ANOVA with target presence (two levels: absent vs. present) and probe location (two levels: singleton distractor vs. non-singleton distractor) as factors. As expected, a main effect of probe location was observed: Letters at the location of the singleton distractor (*M* = 0.24, *SE* = 0.03) were reported less frequently than letters at the non-singleton distractor location (*M* = 0.42, *SE* = 0.03), *F*(1, 33) = 20.33, *p* < 0.001, ${\eta }_{p}^{2}$ = 0.38. Participants also reported more letters at distractor locations overall when the target was absent compared with present, *F*(1, 33) = 30.12, *p* < 0.001, ${\eta }_{p}^{2}$ = 0.48. Because letters at the target location are more frequently reported than those at the distractor locations (both singleton and non-singleton), it is unsurprising that reports of letters at non-target locations are more frequent when the target is absent. Finally, target presence and probe location did not interact, *F* < 1, *BF*_*01*_ = 20.48, indicating that the presence versus absence of a target did not affect the degree to which singleton distractors were suppressed.

### Interim discussion

A suppression effect was observed in our modified multiframe letter-probe paradigm, with lower accuracy for letter probes presented at the location of the singleton distractor compared with non-singleton distractors. Our experiment replicates findings from previous work showing proactive suppression, validating this new version of the experiment, and confirming our first prediction.

We also observed a suppression effect in both target-absent and target-present trials. To our knowledge, this is the first time it has been shown that the amount of singleton distractor suppression is unaffected by any competition between this distractor and a target. These results are consistent with the signal suppression hypothesis, which stipulates that task-dependent control signals can modulate attentional capture via featural gain mechanisms in a *proactive* fashion (which could occur either before or after search display onset, but always before the first shift of attention; see Luck et al., 2021).

## Experiment 2

With our paradigm now validated, we addressed our main question of whether singleton distractor suppression is constrained to a particular feature (i.e., a specific colour; Gaspelin & Luck, 2018b; Vatterott et al., 2018; Won et al., 2019) or if can be applied more broadly to a dimension (i.e., any different-coloured distractor). In Experiment 2, we employed the multiframe letter-probe paradigm in a task where two target and distractor colours swapped randomly (i.e., displays with red search items and a green singleton distractor or with green search items and a red singleton distractor). Previous work from Ma and Abrams (2023a) observed a suppression effect using an analogous colour manipulation, but this may have been due to the shape prevalence task resulting in observers adopting a distinctive search strategy (see *Introduction*). In Experiment 2, observers searched for a feature-defined item, as in Gaspelin and Luck (2018b), who did not observe a suppression effect with an equivalent colour assignment. Our question was whether we could confirm this observation, or whether our multiframe procedure might be sufficiently sensitive to reveal the presence of residual probe suppression at singleton locations.

As in Experiment 1, we investigated whether any such suppression is modulated by the presence or absence of the target. In addition, we also tested whether suppression might be modulated by intertrial priming. Previous research has shown that distractor interference is reduced when salient distractor and search colours remain the same across trials compared with when they are swapped: the singleton distractor interferes with search less when its colour is repeated compared with when its colour is not repeated (De Tommaso & Turatto, 2019; Graves & Egeth, 2015; Vatterott & Vecera, 2012). These findings suggest that suppression can be more easily applied on repeat trials. In this case, evidence of probe suppression at singleton locations might be more pronounced on these trials.

### Method

#### Participants

Thirty-four (34) participants completed Experiment 2 (age: *M* = 28.35 years; *SD* = 10.41 years; 21 women and 13 men). All participants had a mean accuracy within 2.5 standard deviations from the overall mean on the target-counting task (proportion correct: *M* = 0.82, *SD* = 0.11) and therefore no participants were excluded from the sample.

#### Stimuli and procedure

The stimuli and procedure for Experiment 2 were the same as those of Experiment 1 except that now the colour of the singleton distractor and search items (either red or green) were determined randomly for each display and no longer a between-subject factor (see Fig. 1C). Experiment 2 was also conducted online. A constant target shape was used for each participant (circle vs. diamond, determined between subjects), but counterbalancing was not split equally in Experiment 2 due to a coding error. Instead, 25 participants had a circle target and nine participants a diamond target. Overall accuracy was nonetheless similar in both groups, *t*(13.29) = 0.12, *p* = 0.90, *BF*_01_ = 6.41.

#### Analysis

Analyses were the same as Experiment 1, apart from an additional repeated-measures ANOVA, with colour priming (repetition vs. swap) and probe location (non-singleton vs. singleton) as factors.

### Results

Participants reported an average of 1.86 letters per probe frame in Experiment 2, and 1.45 (78%) of these letters were present in the probe array. Participants reported slightly more letters when the target was present than when the target was absent (*M* = 1.46 vs. *M* = 1.45), but this difference was not significant, *t*(33) = 0.4, *p* = 0.69, *d*_*z*_ = 0.07, *BF*_*01*_ = 20.83.

On target-present trials, participants were most likely to report probes that appeared on the target (*M* = 0.69, *SE* = 0.03) compared with the singleton distractor (*M* = 0.24, *SE* = 0.03) and non-singleton distractor (*M* = 0.26, *SE* = 0.03), as reflected by a main effect of probe type, *F*(2, 66) = 207.25, *p* < 0.001, ${\eta }_{p}^{2}$ =0.86. Planned paired *t*-tests showed that letters at target locations were reported more frequently than both distractor types (singleton: *t*(33) = 15.06, *p* < 0.001, *d*_*z*_ = 2.58; non-singleton: *t*(33) = 14.94, *p* < 0.001, *d*_*z*_ = 2.56). However, and in contrast to Experiment 1, no probe suppression effect was observed, as there was no reduction in the frequency of probe letter reports at singleton compared to non-singleton distractor locations, *t*(33) = 1.51, *p* = 0.14, *d*_*z*_ = 0.26, *BF*_*01*_ = 7.80.

To examine the effect of target presence on probe suppression, we conducted a two-way ANOVA with probe location (non-singleton vs. singleton) and target presence (present vs. absent) as factors. A significant interaction between both factors was observed, *F*(1, 33) = 4.24, *p* = 0.048, ${\eta }_{p}^{2}$ = 0.11: When the target was absent, probe reports were slightly more frequent for letters at the singleton compared with the non-singleton location (*M* = 0.37, *SE* = 0.03 vs *M* = 0.36, *SE* = 0.03), whereas an opposite non-significant tendency was observed for target-present trials, as reported above (*M* = 0.24, *SE* = 0.03 vs. *M* = 0.26, *SE* = 0.03; see also Fig. 2B). Given that neither of these differences were significant (target absent: *t*(33) = 1.39, *p* = 0.18, *d*_*z*_ = 0.24, *BF*_*01*_ = 10.00; target present: see above), we do not consider it any further.

We also confirmed that the effect of repetitions versus swaps of target/singleton distractor colours between the final frame of each trial and the immediately preceding frame did not affect probe suppression. A repeated-measures ANOVA (collapsed over target presence) with the factors colour priming (repetition vs. swap) and probe location (non-singleton vs. singleton) as factors did not result in any main effect or interaction involving the factor colour priming, both *Fs* < 1, both *BF*_*01*_s > 20*.* Finally, we observed no evidence of learning effects (see Online Supplementary Material), though there was marginal evidence for initial attentional capture of the singleton during Block 1 (in line with previous research, e.g., Vatterott & Vecera, 2012).

### Interim discussion

As predicted, no suppression effect was observed when target and singleton distractor colours swapped randomly across displays, confirming the results of Gaspelin and Luck (2018b). This was the case for both target-absent and target-present trials, suggesting again that target presence does not modulate distractor suppression (if anything, there was a trend towards attentional capture by singleton distractors on target-absent trials). We also did not find any differential effects of intertrial priming. If suppression can be applied more readily when target and singleton distractor colours are repeated than when they change, residual distractor suppression may have been observed specifically for change trials, but this was not the case.

Given these results, it might seem tempting to conclude that it is only possible to suppress specific features, in line with a first-order suppression account (Gaspelin & Luck, 2018b). However, the absence of distractor suppression in Experiment 2 might be because only two colours were employed, so that target and salient distractor colours either repeated or were swapped across trials. It remains possible that the variability in colour space was insufficient to encourage feature-unspecific second-order suppression. This type of suppression may also have interfered with target processing, since the colour of the target matched the colour as the singleton distractor in the preceding display in half of all displays (see also Wirth et al., 2023). In other words, the probability of the salient distractor colour in a trial being the target colour in other trials is relatively high. In this context, it might be more effective to ignore the colour swapping entirely and treat all items as equally relevant rather than suppressing the uniquely coloured item below baseline. Experiment 3 was designed to test this possibility by substantially increasing the number of possible target and distractor colours.

## Experiment 3

In Experiment 3, we employed eight different possible colours for target/non-singleton distractors and the singleton distractor. The colour of the search items (target and non-singleton distractors) and singleton distractor were chosen at random on each trial, with the constraint that they could never be the same. If second-order suppression can be applied when the variability and unpredictability of the colour singleton and non-singleton search display items is increased, decreasing the probability of any one colour being the potential target colour, a suppression effect should now be observed, with probe letter reports less frequent at singleton relative to non-singleton distractor locations. Such a result would demonstrate that second-order suppression can be applied in tasks where observers search for a feature-defined target (here, shape). Experiment 3 was run in the lab rather than online, because the increased colour variability made it more difficult to control and equate the luminance of target and singleton distractor colours. This can be done much more reliably in the lab than in an online experimental setting.

### Methods

#### Participants

Thirty-seven (37) participants completed Experiment 3. One participant was removed due to having a mean accuracy above 2.5 standard deviations from the overall mean on the target counting task, and two were removed for not completing the experiment, leaving 34 participants in the final sample (age: *M* = 30.29 years; *SD* = 8.54 years; 22 women and 12 men). Overall counting task mean for the final sample was 86% (*SD* = 10%).

#### Stimuli and procedure

The stimuli and procedure for Experiment 3 were the same as for Experiment 2 except for the following changes. This experiment was conducted in a laboratory setting at Birkbeck, University of London, rather than online. Consent was received on paper and no longer embedded in the experiment. Participants completed the task in a dimly lit room on a 24-in. BenQ monitor (1,920 × 1,080 screen resolution) attached to a SilverStone PC. The size of stimuli in Experiment 3 were similar as those in Experiment 1 and Experiment 2 (in visual angles), with the following changes: viewing distance was now 80 cm (rather than approximately 60 ms), and stimuli were roughly 1.2 times larger than those in Experiments 1 and 2 (though the exact measures are not possible to obtain as both previous experiments were conducted online). The experiment was programmed using E-prime 3.0 software (Psychology Software Tools, Pittsburgh, PA, USA). The number of trials was also doubled, with 200 trials divided to five experimental blocks (ten experimental blocks for two subjects), to take into consideration the increased number of colours used.

Instead of only two colours (Experiment 2), eight colours were used: (1) Brown (luminance: 40 cd/m^2^; CIE coordinates: 0.476/0.462; HEX colour code: #834d00). (2) Khaki green (40.3 cd/m^2^; 0.407/0.515; #5f5a00). (3) Forest green (40.3 cd/m^2^; 0.332/0.576; #2b6200). (4) Teal (40.0 cd/m^2^; 0.255/0.328; #006166). (5) Blue (39.8 cd/m^2^; 0.199/0.253; #005d88). (6) Purple (40 cd/m^2^; 0.216/0.100; #9600f6). (7) Pink (40.1 cd/m^2^; 0.333/0.165; #bc00a3). (8) Red (39.9 cd/m^2^; 0.496/0.260; #CA0055). The target/non-singleton distractors and singleton distractor colours were chosen randomly from the eight possibilities for each visual search presentation (Fig. 1D), with the constraint that the same colour was not selected for both. The analysis conducted was the same as Experiment 1. Unlike Experiment 2, we did not analyse full colour-priming effects between the previous search display and the probe display, as the inclusion of eight colours resulted in too few full repetition and full swap trials to allow for a meaningful analysis of this factor. Instead, we examined only search colour-to-salient distractor colour repetitions. If the relevant search colour (i.e., the majority colour in both target-absent and target-present displays) was enhanced when it is present in the previous display, the likelihood of reporting a singleton of that colour in the subsequent probe display should be increased.

### Results

Participants reported an average of 2.37 letters per probe frame in Experiment 3, and 1.87 (79%) of these letters were present in the probe array. As in Experiments 1 and 2, participants reported a roughly equal number of correct letters regardless of whether the target was present (*M* = 1.86, *SE* = 0.11) or absent (*M* = 1.87, *SE* = 0.11), *t*(33) = 0.41, *p* = 0.68, *d*_*z*_ = 0.07, *BF*_*01*_ = 31.25.

Increasing the number of possible colour combinations produced results that were similar to Experiment 1 (Fig. 2C). When the target was present, letter report frequencies were highest for probes that appeared in the target location (*M* = 0.80, *SE* = 0.02), lower in the non-singleton distractor location (*M* = 0.38, *SE* = 0.04), and lower still in the singleton distractor location (*M* = 0.30, *SE* = 0.03). In line with this observation, the main effect of probe location was significant,* F*(2, 66) = 187.24, *p* < 0.001, ${\eta }_{p}^{2}$ = 0.85, as were the comparisons between each pair of locations, all *t*s > 3.77, all *p*s < 0.002, all *d*_*z*_*s* > 0.65 (two-tailed; corrected for multiple comparisons using Bonferroni), including the difference between singleton and non-singleton probe reports, *t*(33) = 3.78, *p* = 0.002, *d*_*z*_ = 0.65. Thus, a clear singleton distractor suppression effect was observed.^4^ To test whether target presence modulated this effect, a two-way ANOVA was run that included probe location (non-singleton vs. singleton distractor) and target presence (absent vs. present) as factors. Like Experiment 1, the main effect of probe location was significant, *F*(1, 33) = 12.36, *p* = 0.001, ${\eta }_{p}^{2}$ = 0.27. A larger suppression effect was observed when the target was present compared with when it was absent (*ΔM* = 0.08 vs. *ΔM* = 0.03), reflected by a significant interaction between both factors, *F*(1, 33) = 7.13, *p* = 0.01, ${\eta }_{p}^{2}$ = 0.18. We note, however, that a suppression effect was observed for both conditions, both *p*s < 0.05. We also examined whether second-order suppression became stronger over time and observed no change in the suppression effect across the duration of the experiment (see Online Supplementary Material). This absence of suppression-related learning effects is similar to Experiment 2.

An additional exploratory analysis was conducted to examine whether probe suppression effects are modulated by the relationship between the search target colour in the previous display and the salient distractor colour in the probe display. If the absence of an overall suppression effect in Experiment 2 was due to target feature enhancement from the previous trial (i.e., colour-priming effects), then the suppression effect should disappear when the search colour in the preceding display is the salient distractor colour in the probe display. Approximately 12% of the data was retained (~ 24 trials per participant; collapsed over target presence). A two-tailed paired t-test comparing probe reports at the singleton and non-singleton locations for these trials revealed a probe suppression effect, *t*(33) = 2.75, *p* = 0.01, *d*_*z*_ = 0.47; singleton distractor: *M* = 0.36, *SE* = 0.035; non-singleton distractor: *M* = 0.43, *SE* = 0.034). This shows that suppression of salient distractors was not eliminated by any colour priming from the preceding display, and further supports the conclusion that distractor suppression was not colour-specific, but instead based on the presence of a feature discontinuity.

#### Comparison of experiment 1 and experiment 3

While both Experiment 1 and Experiment 3 produced reliable suppression effects, Fig. 2 suggest that the magnitude of the suppression effect was smaller in Experiment 3. To assess potential differences between both experiments, we first assured an equal number of trials by only including the first half of trials in Experiment 3. The difference between the proportion of probe reports at singleton and non-singleton distractor locations was 0.19 in Experiment 1 but only 0.05 in Experiment 3 (collapsed over target presence). This difference was confirmed to be significant using a Welsh two-sample t-test, *t*(42.32) = 3.13, *p* = 0.003, *d* = 0.76.

Accuracy in the counting task was generally better in Experiment 3 than in Experiment 1 (86% vs. 75%; *t*(59.64) = 3.12, *p* = 0.003, *d* = 0.76), though overall correct probe letter reports were not significantly different between the two experiments (but numerically higher in Experiment 3 (1.83 probes vs. 1.63 probes; *t*(65.71) = 1.31, *p* = 0.19, *d* = 0.31, *BF*_*01*_ = 8.20)). However, better probe report accuracy in Experiment 3 was not observed for all probe locations: For target-present trials, probe report accuracy was better at the target location, Δ = 0.17, and at the singleton distractor location, Δ = 0.10, but similar at non-singleton distractor locations, Δ = −0.02. In line with this observation, a two-way mixed ANOVA for target present trials with experiment (two levels: 1 vs. 3) and probe location (three levels: non-singleton distractor, singleton distractor, target) as factors showed probe accuracy to be overall higher for Experiment 3 (*M* = 0.49, *SE* = 0.03) compared with Experiment 1 (*M* = 0.41, *SE* = 0.02), *F*(1, 66) = 5.91, *p* = 0.018, ${\eta }_{p}^{2}$ = 0.08, and a significant interaction between the two factors, *F*(2, 132) = 7.18, *p* = 0.001, ${\eta }_{p}^{2}$ = 0.10. Bonferroni-corrected comparisons (three contrasts) showed that target location probe reporting was better in Experiment 3 than Experiment 1, *t*(56.17) = 4.58, *p* < 0.001, *d* = 1.11, with an inconclusive difference for singleton distractors, *t*(63.01) = 2.14, *p* = 0.11, *d* = 0.52, *BF*_*01*_ = 1.02, and no difference for non-singleton distractors, *t* < 1, *d* = 0.09, *BF*_*01*_ = 5.06.

### Interim discussion

The critical new result of Experiment 3 was that a clear suppression effect for probe reports at singleton distractor locations was observed when the number of possible search display colours was increased from two to eight. This observation strongly suggests that second-order suppression of salient singleton distractors is possible. It demonstrates for the first time that first-order suppression of feature singletons is not the only option in search tasks where targets are defined by a unique feature as proposed by Gaspelin and Luck (2018b). Our results further demonstrate that global analysis of the search display is not required for second-order suppression, as suggested by Ma and Abrams (2023a), and that suppression is possible when using a strategy that encourages searching for a unique target item. As in Experiment 1, distractor suppression was present both when search displays included a target and when they did not.

Interestingly, distractor suppression was stronger in Experiment 1 than in Experiment 3. This suggests that although second-order suppression is available under certain conditions, first-order suppression applied when singleton distractor colours are constant and predictable is the more effective strategy. It is possible that this type of suppression can be proactively based on a feature-specific “template for rejection” (i.e., a unique constant distractor; Arita et al., 2012), whereas second-order suppression is reactive and depends on the rapid detection of a feature discontinuity in a visual search display (i.e., a unique changing distractor). This possibility should be explored in future research. The fact that performance in the target-counting task and the letter-probe report tasks was better in Experiment 3 is likely to be due the fact that this experiment was run in a laboratory setting rather than online.

## General discussion

Past experience, or selection history, plays an important role in our ability to manage distracting information (e.g., Anderson, 2021; Kim et al., 2022; Luck et al., 2021). While there is strong evidence for experience-based suppression associated with spatial expectations (Huang et al., 2022; Liesefeld & Müller, 2021; Sauter et al., 2018; Theeuwes et al., 2022), investigations of the experience-based suppression of expected distractor features have led to inconsistent conclusions (Gaspelin & Luck, 2018b; Liesefeld et al., 2022; Ma & Abrams, 2023a, b; Won & Geng, 2018). Research originally showed that features need to be predictable and specific for learned distractor suppression to be applied during visual search (first-order suppression, e.g., Gaspelin & Luck, 2018b; Vatterott & Vecera, 2012; Vatterott et al., 2018). Recent evidence, however, has suggested that it is possible to suppress singleton distractors that vary unpredictably on a task-irrelevant dimension (i.e., second-order suppression) under certain circumstances (majority search; Ma & Abrams, 2023a; reduction in capture using the additional-singleton paradigm; Won et al., 2019). However, no studies have so far provided direct evidence for second-order suppression of salient distractors when observers search for a specific target object. In fact, no evidence for suppression was found when the colour of the target/non-singleton distractors and the singleton distractor changed unpredictably, using up to four different colour combinations (Gaspelin & Luck, 2018b). Here, we show for the first time that it is possible to suppress salient singleton distractors when searching for a unique feature-defined target during visual search even when the distracting feature (here, colour) is unpredictable. Importantly, this was the case in the absence of a search strategy that encouraged global analysis (Ma & Abrams, 2023a), and without keeping the task-relevant colour (i.e., the colour of the target and non-singleton distractors) constant (Won et al., 2019). We also showed that this type of suppression was applied to singleton distractors regardless of whether the target item was present, suggesting that it did not depend on a direct competition between a target and the singleton distractor. These observations demonstrate that distractor suppression can be applied in a more flexible manner during attentional guidance than previously assumed.

Our results raise the obvious question why second-order suppression was observed in Experiment 3, but not in previous studies using the letter-probe paradigm (Gaspelin & Luck, 2018b). An important difference is that in this earlier work, a small number of target/distractor colour configurations was employed. Because each distractor singleton colour also appeared as frequently as the colour of the target, first-order suppression of these colours would have been an ineffective search strategy. With two possible target/distractor colours, participants may have instead activated search templates for both target colours (see Grubert & Eimer, 2016, and Irons et al., 2012, for evidence for multiple colour-specific search templates).^5^ Multiple colour target templates may be incompatible with applying suppression in a generalised feature-unspecific fashion to colour singleton distractors. Instead, singleton and non-singleton distractors would be considered equally likely to be potential targets, which is why no difference in probe report accuracy at these locations was observed in Experiment 2 (see also Gaspelin & Luck, 2018b; Experiments 1 and 2). However, in a corresponding eye-tracking experiment (Experiment 3 of Gaspelin & Luck, 2018b), first eye movements were more likely to be directed toward the singleton distractor than the non-singleton distractors, suggesting that the singleton captured overt attention to some degree, and that eye movements may be more sensitive than probe letter reports to rapid salience-based attentional capture. Another difference between our task and that of Gaspelin and Luck (2018b) is that we required participants to count the number of displays with a target instead of reporting the target for every display. It is unclear, however, why this response manipulation would impact attentional mechanisms since each individual display could only contain a single target object (in contrast to the majority search task employed by Ma & Abrams, 2023a). Furthermore, previous electrophysiological research suggests that attention is deployed similarly in multiple frame procedures relative to single frames (with a slight onset delay for the first display; Aubin & Jolicoeur, 2016).

A different possible explanation for differences in probe reports at the singleton and non-singleton locations is global target-feature enhancement, which would also facilitate the non-singleton distractors because they possess a target feature (here, colour). In support of this view, Oxner et al. (2023) demonstrated that the suppression effect disappears when the non-singleton object and target colours are different, and that non-singleton probe recall rises in a graded fashion with colour similarity to the target. In the present work, results from Experiment 1 and Experiment 2 could be explained by this account: The suppression effect in Experiment 1 appears to be driven by more frequent probe reports for the non-singleton items, with singleton probe reporting similar across both experiments. This could suggest that non-singleton locations in Experiment 1 were enhanced because they possessed a constant target feature (colour), which was not the case in Experiment 2 where the colours swap randomly. However, this interpretation cannot explain the results of Experiment 3, where the probability of reporting letters was lower at the singleton relative to non-singleton locations, even though search colour (target and non-singleton items) changed between most displays. It is highly unlikely that global target-feature enhancement can be applied to eight different colours, leaving second-order suppression the more parsimonious explanation for these results. While we cannot completely dismiss the possibility that global feature enhancement may contribute to differences between singleton and non-singleton probe locations, interpretations of performance differences between experiments should be made with caution, as they can be susceptible to individual differences. Moreover, a different pattern of results has been observed in previous similar studies: When search and singleton colours were kept constant (Gaspelin et al., 2015, Exp. 4) or swapped randomly (Gaspelin & Luck, 2018b; Exp. 1), while all other parameters were identical, non-singleton location probe report frequencies were very similar (0.33 and 0.36, respectively). Future research is needed to determine the relative contribution of target feature enhancement and distraction inhibition during visual search (see also Chang & Egeth, 2019).

In Experiment 1 and Experiment 3, suppression was applied to salient distractors regardless of target presence. These results are in line with theories of proactive suppression (e.g., Gaspelin & Luck, 2018a; Gaspelin et al., 2015; Sawaki & Luck, 2011), which suggest that singleton suppression relies on control processes that are activated prior to the first shift of attention (either before or after search display onset). They are also consistent with work demonstrating that target and distractor processing are two independent processes (e.g., Chang & Egeth, 2019). This is in line with electrophysiological studies of salient distractor suppression, which have shown that the allocation of attention to the target item (reflected by the N2pc component, occurring approximately 200–300 ms following stimulus onset) occurs after distractor suppression (reflected by an early P_D_ component, occurring around 150–300 ms after stimulus onset, e.g., Drisdelle & Eimer, 2021; Gaspar & McDonald, 2014; Gaspelin & Luck, 2018a; Sawaki & Luck, 2010). Because participants had no way of knowing at the beginning of a trial whether a target would be present or absent in any given display, they were not able to strategically apply any distractor suppression differentially depending on whether a target was present or not (see also Moher, 2020, Supplementary Material). However, previous event-related potential (ERP) research also suggests that suppression might be affected by the presence of a target. An enhanced P_D_ was observed when the target was present, and this was interpreted as a stronger need for suppression in target-present displays to facilitate target selection (Drisdelle & Eimer, 2021; but see Tam et al., 2022). If this were the case, probe suppression effects at the location of the singleton distractor should be stronger when a target is present rather than absent. There was some evidence for such a pattern in Experiment 2 and more notably in Experiment 3, indicating that suppression might have been slightly stronger in the presence of a target, when the need to avoid capture by the salient distractor was higher. It is possible that the presence of a target was registered pre-attentively on some occasions, early enough to affect the suppression of the distractor.

In summary, our results show, for the first time, that suppression can be applied to singleton distractors when their salient feature varies unpredictably in the context of feature-defined target search (as opposed to majority search; Ma & Abrams, 2023a). Letter probes at the colour singleton distractor location were reported less frequently than those at the non-singleton distractor locations, not only when the singleton colour remained constant throughout, but also when it was chosen randomly from a set of eight different colours. These observations support accounts of *second-order feature suppression*, which stipulate that items can be suppressed based on local discontinuities within a given feature dimension (Gaspelin & Luck, 2018b). They demonstrate that the suppression of salient distractors does not always depend on the availability of information about their features that is acquired through learning. Under certain circumstances, suppression can also be applied in feature-nonspecific fashion.

## Supplementary Information

Below is the link to the electronic supplementary material.

Supplementary file1 (DOCX 1362 KB)

## Data Availability

All data have been made publicly available on Figshare and can be accessed at 10.6084/m9.figshare.24851442.v1.

## Footnotes

1 Multiple frame procedures have been previously used in studies measuring event-related potentials (ERPs) to maximise the number of search displays. In electrophysiological research, simplifying the task can also minimise electrical activity elicited by other processes that might not be relevant to the scientific question (i.e., evoked potentials associated with response execution; Aubin & Jolicoeur, 2016; Drisdelle & Eimer, 2021, 2023; Drisdelle & Jolicoeur, 2018; Drisdelle et al., 2017; Maheux & Jolicœur, 2017).

2 A technical error led to the removal of trials where the letter A was recorded using a keyboard as this response included key presses for both the letter A and the letter H. This affected trials where the keyboard was used for 12 participants (8% of trials). This error was corrected in subsequent participants and experiments.

3 All visual angle measurements reported depend on participants being at 60 cm from the monitor. Because they used their arm’s length as a rough estimation, these values are all approximations.

4 Because Experiment 3 was considerably longer than the first two experiments (200 vs. 100 trials), it is possible that participants only developed the ability to suppress singleton distractors in the final 100 trials. To test this possibility, we calculated the suppression effect in the first 100 trials of Experiment 3 (collapsed over target-absent and target-present trials). Probe report accuracy was significantly lower at the location of the singleton distractor (*M* = .367) compared with the non-singleton distractor (*M* = .414), *t*(33) = 3.04, *p* = .005, *d*_*z*_ = 0.52.

5 Even when Gaspelin and Luck (2018b) increased the number of configurations from two to four, the colours were paired together when swapped (i.e., green-pink, pink-green, blue-orange, orange-blue), resulting in a small number of possible colour configurations.

## Code availability

N/A.

## Declarations

### Open practices statement

All data have been made publicly available on Figshare and can be accessed at 10.6084/m9.figshare.24851442.v1. These studies were not preregistered.
