# Supplementary material for: Unpredictable singleton distractors in visual search can be subject to second-order suppression
Source: Atten Percept Psychophys. 2025 Feb 26;87(3):832–47. doi: 10.3758/s13414-025-03028-3 (PMC11965257; doi:10.3758/s13414-025-03028-3)
Supplement: Supplementary file 1 — Supplementary file1 (DOCX 1362 KB) [file 13414_2025_3028_MOESM1_ESM.docx]

**Supplementary material**

**Experience or learning-based effects in second-order suppression**

Several studies have shown that, for first-order suppression, singletons initially capture attention, with suppression building after a small number of trials with a consistent coloured distractor (i.e., Vatterott & Vecera, 2012; Gaspelin & Luck, 2018). Interestingly, these experiments did not observe second-order suppression (i.e., Gaspelin & Luck, 2018), though have acknowledged that participants could eventually learn to suppress singletons based on salience alone with extensive practice or training. Here we explore whether our experimental design (multiple frame procedure), which does not require a response after every display (and therefore includes more search displays in less time than most traditional search tasks), might allow us to observe any learning effects.

**Learning-based effects in Experiment 2 (2 mixed colours):**

It is possible that, in Experiment 2 (2 mixed colours), aggregating the data across trials masked early capture (in the first block) and late second-order suppression (in the last block). To test this possibility, we conducted a 2 (distractor type: singleton vs nonsingleton distractor) by 5 (block: 1, 2, 3, 4, 5) repeated-measures ANOVA. As expected, we observed no evidence for an overall suppression effect (main effect of distractor type: *F* < 1). We also observed no effects according to block number (main effect: *F*(4, 132) = 1.2, *p* = .31, $\eta_{p}^{2}$ =.04; interaction with distractor type: *F*(4, 132) = 1.44, *p* = .22, $\eta_{p}^{2}$ =.04), suggesting that distractor processing did not change over time. We nonetheless examined whether capture occurs early on (Block 1) and if suppression was observed with training (Block 5) directly. Paired t-tests showed a trend for capture in Block 1, *t*(33) = 2.01, *p* = .053, with probes at the singleton distractor (.34, *SE* = .03) being reported more frequently than probes at the nonsingleton distractor (.29, *SE* = .03), and for suppression in Block 5, *t*(33) = 1.43, *p* = .16 (singleton: *M* = .30, *SE* = .03; nonsingleton: *M* = .32, *SE* = .03), though neither reached significance. These (non-significant) trends are seen in Figure 1.

**Figure 1**

*Difference between singleton and nonsingleton probe reports in Experiment 2 (2 mixed colours)*


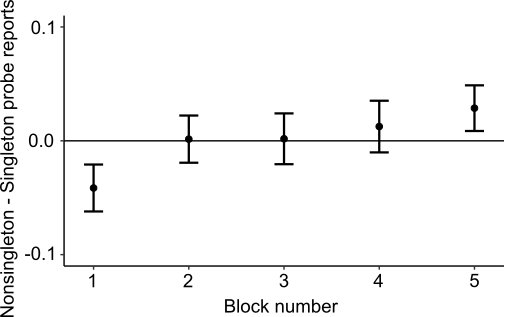


*Note:* Changes over Block (1 to 5) in probe reporting at the nonsingleton and singleton locations (collapsed over target presence). A negative value indicates that probes at the singleton location were reported more frequently (capture) and a positive value indicates that probes at the nonsingleton location were reported more frequently (suppression). Error bars represent the standard error of the mean.

**Learning-based effects in Experiment 3 (8 mixed colours):**

We also explored whether experience alters the suppression effect over time in Experiment 3 (8 mixed colours). We conducted a similar analysis as for Experiment 2 but split each block into two halves because they were twice as long (40 instead of 20 trials per block). As expected, probes at the singleton location were reported less frequently than at the nonsingleton location overall (main effect: *F*(1, 33) = 12.35, *p* = .001, $\eta_{p}^{2}$ = .27). A main effect of block was also observed, *F*(9, 297) = 2.41, *p* = .012, $\eta_{p}^{2}$ =.07, which is driven by an overall increase in probe report accuracy over time (see Figure 2). This is confirmed by a significant correlation between block number and probe report accuracy (collapsed over singleton and nonsingleton distractor locations), *r* = .75, *p* = .013. The suppression effect did not change over time (interaction between block and probe location: *F* < 1).

**Figure 2**

*Suppression effect by block in Experiment 3 (8 mixed colours)*


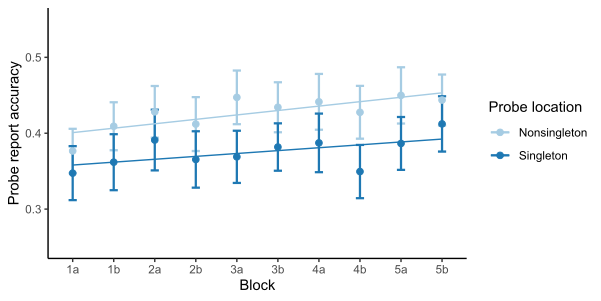


*Note:* Probe report accuracy in Experiment 3 is shown for Probe location (nonsingleton and singleton distractors; collapsed over target presence) and Block (1 to 5) separately. Each block was separated into 2 parts, a and b, so that block length is comparable to Experiment 2 (20 trials per block). Error bars represent the standard error of the mean.

**References:**

Vatterott, D. B., & Vecera, S. P. (2012). Experience-dependent attentional tuning of distractor rejection. *Psychonomic bulletin & review*, *19*, 871-878.

Gaspelin, N., & Luck, S. J. (2018). Distinguishing among potential mechanisms of singleton suppression. *Journal of Experimental Psychology: Human Perception and Performance*, *44*(4), 626–644.
